# Insights from Real-World Evidence on the Use of Inhalers in Clinical Practice

**DOI:** 10.3390/jcm14041217

**Published:** 2025-02-12

**Authors:** Myriam Calle Rubio, Pedro José Adami Teppa, Juan Luis Rodríguez Hermosa, Miriam García Carro, José Carlos Tallón Martínez, Consolación Riesco Rubio, Laura Fernández Cortés, María Morales Dueñas, Valeria Chamorro del Barrio, Rafael Sánchez-del Hoyo, Jorge García Aragón

**Affiliations:** 1 Pulmonology Department, Instituto de Investigación Sanitaria del Hospital Clínico San Carlos (IdISSC), 28040 Madrid, Spain; mcal01@ucm.es (M.C.R.); pedrojose.adami@salud.madrid.org (P.J.A.T.); jorge.garcia.aragon.jg@gmail.com (J.G.A.); 2 Department of Medicine, School of Medicine, Universidad Complutense de Madrid, 28040 Madrid, Spain; 3 CIBER de Enfermedades Respiratorias (CIBERES), 28029 Madrid, Spain; 4 Farmacy Department, Hospital Clínico San Carlos, 28040 Madrid, Spain; 5 Unidad de Soporte Metodológico a la Investigación, Servicio de Preventiva, Instituto de Investigación Sanitaria del Hospital Clínico San Carlos (IdISSC), 28040 Madrid, Spain

**Keywords:** inhaler, peak inspiratory flow, critical inhaler errors, compliance, adherence, inhaler handling-related knowledge

## Abstract

**Background:** Despite the ongoing innovations and the availability of numerous effective inhaled treatment options, achieving optimal disease control in most patients frequently remains disappointing. Unfortunately, although inhaled therapy is the cornerstone of respiratory disease management, the selection of the most appropriate inhaler is still overlooked or underestimated by some healthcare professionals, and inhaler misuse remains a significant challenge in managing chronic respiratory diseases which directly influences patients’ quality of life, clinical outcomes, and risk of disease progression. **Materials and Methods:** This is a unicentric, observational, cross-sectional study designed to evaluate the inhaled therapy prescribed in hospitalized patients and to analyze device changes after hospitalization, as well as the factors associated with these changes. A single face-to-face visit was performed during the patient’s hospitalization, where the inhaled therapy used prior to hospitalization was evaluated: technique (critical errors), compliance (TAI questionnaire), maximum peak inspiratory flow [PIF (L/min)], and level of inhaler handling-related knowledge. A binary logistic regression model was used to explore the association between changing device at discharge and the other independent variables **Results:** The inhaler most used during hospitalization was the metered-dose inhaler (MDI) with a chamber (51.9% of patients), with the dry powdered inhalers (DPI) being the inhalers used in 43% of maintenance inhaled therapies in the community setting prior to hospitalization. In addition, 90% of patients showed a maximum PIF ≥ 30 L/min, and 35.6% performed critical inhaler errors. These patients had statistically significantly lower maximum PIF values (52.1 L/min in patients with critical inhaler errors vs. 60.8 L/min without critical inhaler errors; *p* > 0.001) and were more likely to exhibit poor inhaler compliance compared to those without critical errors (50.5% vs. 31.0%, respectively). More than half of the patients who used MDI with spacer chamber made critical inhaler errors; 69.9% showed regular or poor treatment adherence, although 75.6% demonstrated good knowledge about inhaler handling. Only in 27% of the patients did the healthcare professional change the type of inhaler after hospitalization within clinical practice. The medical and nursing staff responsible for the patient’s hospitalization were not informed of the assessment carried out in the study. The probability of not performing a device change at discharge was lower in patients with previous at-home treatment with combined inhaled therapy with LABA + ICS (OR 0.3 [0.18–0.83], *p* = 0.016) and in patients under triple inhaled therapy (OR 0.3 [0.17–0.76], *p* = 0.007). No significant differences were observed in inhaler changes when considering the frequency of critical inhaler errors, inhaler handling-related knowledge or maximum PIF values. **Conclusions:** Our study highlights the urgent need for a more personalized inhaler selection and consistent monitoring by healthcare professionals to minimize inhaler misuse, increase treatment compliance and adherence, and improve disease management outcomes. It is essential to provide training and promote the role of nursing in the evaluation and education of inhaled therapy. Additionally, the use of standardized approaches and tools, such as the CHECK DIAL, is crucial to facilitate the adaptation of devices to patients’ needs.

## 1. Introduction

Chronic diseases are the leading cause of deaths worldwide, with chronic respiratory diseases among those causing the highest rates of mortality and morbidity [1,2]. Chronic respiratory diseases affect 45 million people worldwide, a 39.8% increase since 1990, according to an analysis from the Global Burden of Disease Study [3].

The cornerstone of respiratory disease management is the use of inhaled treatments, which enable a rapid and targeted delivery of drugs to the lungs while minimizing systemic exposure and potential side effects [4,5]. In fact, inhaled therapy is among the most frequently prescribed treatments, being the choice of the inhaler device considered just as important as selecting the most appropriate drug [4]. Errors in inhalation technique are linked to worse disease control and increased healthcare resource utilization, including higher rates of hospitalizations and emergency room visits [6]. A systematic review found that incorrect inhalation techniques have not significantly improved over the past 40 years, with only 31% of patients demonstrating proper technique [7]. However, this critical aspect is often overlooked or underestimated by healthcare professionals in both primary and secondary care [4].

Despite the advancements in inhaler device technology, the “perfect inhaler” does not exist, with dry powdered inhalers (DPIs) and metered-dose inhalers (MDIs) remaining as the two most widely prescribed inhaler types [8]. DPIs are typically associated with fewer critical errors and, due to their lower carbon footprint, are considered more eco-conscious devices [9,10]. Selecting the most appropriate inhaler, aligned with the patient’s ability to use it correctly and the inhaler reliability in delivering medication, significantly influences treatment adherence with prescribed timing, dosage, and frequency and, ultimately, impacts disease outcomes [11,12]. Thus, it is important for healthcare professionals to consider treatment strategies that optimize both patient health and environmental sustainability. To achieve a balance, it becomes essential the assessment of patient characteristics and the ongoing education and training on inhalation techniques.

Factors such as cognitive function, manual dexterity, and peak inspiratory flow (PIF) should be carefully evaluated when selecting an inhaled delivery system, as these considerations have been shown to enhance treatment adherence and efficacy [13]. Regular education sessions, including physical demonstrations of proper device use and technique correction, may improve symptom control and allow for long-term dose reduction [14].

Considering the relevance of these topics in real clinical practice, it is essential to collect real-world evidence on most commonly used inhalers, highlighting patterns of misuse, compliance, and adherence, alongside relevant clinical information. This evidence will be critical for the optimization of inhaler selection and usage and, ultimately contribute to improved clinical outcomes, reduce side effects, enhance patient adherence, and decrease the overall healthcare costs. This study evaluates the characteristics of patients hospitalized while using inhaled therapy. Moreover, we also analyzed the frequency of critical inhaler errors and adherence to inhaled treatment prior to hospitalization, as well as the changes made to inhaler devices at discharge and the factors associated with these changes.

## 2. Materials and Methods

### 2.1. Study Overview

The AIRE project is a unicentric, observational, cross-sectional study conducted between March 2023 and March 2024. The study population consisted of hospitalized patients who were prescribed inhaled therapy during their hospitalization. The patient recruitment was prospective, and each week was consecutively included data of inpatients under inhaled therapy during hospitalization across different medical inpatient services. The inclusion criteria were patients aged ≥18 years who were receiving inhalation therapy during hospitalization, while the exclusion criteria were patients with severe cognitive impairment or those unable to complete inhalation technique testing. A cross-sectional review of treatments was performed every week using the electronic prescription program for hospital admission across various inpatient departments (internal medicine, pulmonology, geriatrics, and others [cardiology, endocrinology]).

After patient selection, a single face-to-face visit was performed during the patient’s hospitalization by nurses specializing in inhaled therapy education from the pulmonology service. The data collected were concurrent for clinical information (performed during the single visit) and retrospective for the assessment of patient history or tests performed in the previous year.

This study was designed to evaluate the suitability of inhaled therapy in hospitalized patients, both with and without a history of inhaler use, prior to hospitalization, as well as their inhalation technique (critical inhaler errors), adherence to previously prescribed inhaled treatments, the frequency of inhaler changes after hospitalization, and the factors associated with these changes.

The project was approved by the Ethics Committee of the Clinical Hospital of San Carlos (CI:23/069-O_M_NoSP). All participants signed an informed consent prior to the enrollment in the study, in accordance with the principles of the Declaration of Helsinki and Spain’s new Organic Law 3/2018 on the Protection of Personal Data and the Guarantee of Digital Rights, effective since 7 December 2018.

### 2.2. Data Source and Patient Selection

**Figure 1:**
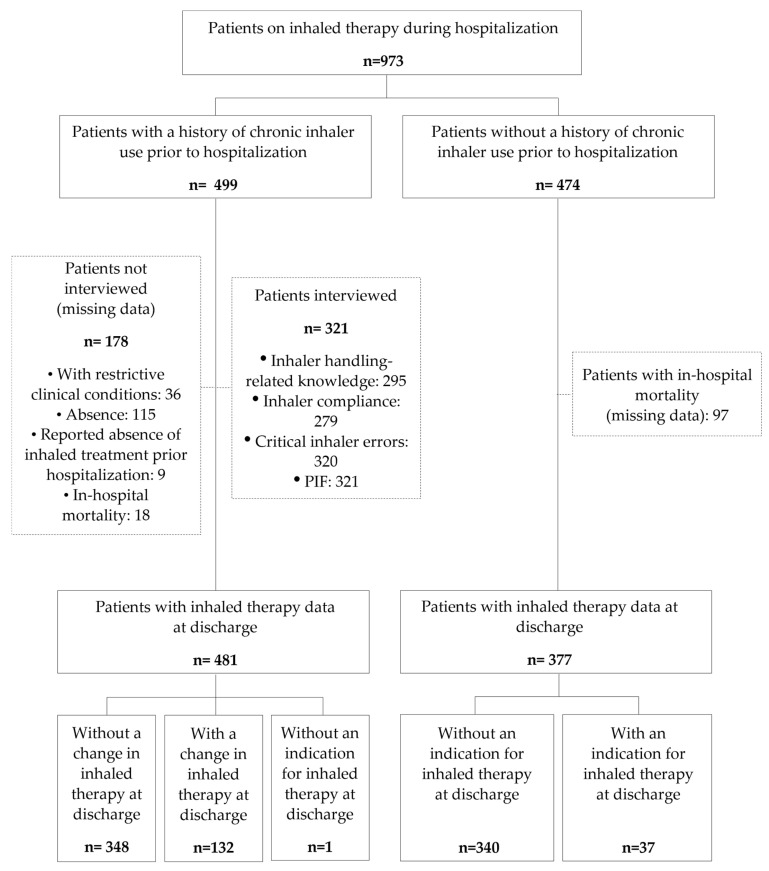
Flow chart of the patients included and evaluated in the AIRE study. PIF, peak inspiratory flow.

Hospitalized patients receiving inhaled pharmacotherapy (bronchodilators and anti-inflammatory drugs) were identified through their electronic prescription history during the weekly cross-sectional review of treatments at the Clinical Hospital of San Carlos (Figure 1). The source of information on the prescription of inhaled therapies during hospitalization was the in-hospital e-prescribing program, FarmaTools^®^, version 3.0.

### 2.3. Data and Clinical Variables Collected

Data regarding inhalers prescribed during the hospitalization period were collected from FarmaTools^®^. Clinical information was provided by the Selene Plus^®^ program and the Horus^®^ Primary Care program, including comorbidities (Charlson index [15]), current smoker status, history of hospitalizations, history of antibiotic/corticosteroid courses for respiratory diseases, cause of hospitalization, responsible inpatient service, and other relevant data (e.g., in-hospital mortality rate and 90-day post-discharge mortality rate). A total of 973 patients were included in the study, of whom 410 were male (41.9%) with a mean age of 76.4 (12.4) years, and 65.7% had a Charlson index ≥ 2.

For patients with a history of prior inhaler use, information on their at-home inhaled therapy before hospitalization (therapeutic class, inhaler type and number, posology, prescriber, trainer, and treatment duration) was collected from the Single Prescription Module (MUP) program, a tool that provides a unified and comprehensive pharmacotherapeutic history of the patient available in Madrid.

During a face-to-face visit conducted during the patient’s hospitalization, additional data were collected, including details about compliance and inhalation technique (critical inhaler errors in dry powder device users were failure to exhale before inhalation and insufficient inspiratory effort, and in metered-dose inhalers (MDIs) or SMI users, it was activation of the inhaler before inhalation or breathing in too quickly) with at-home inhaler treatment, assessed using the TAI questionnaire [16], and measurement of maximum PIF (L/min). Maximum PIF was evaluated with the In-Check DIAL G16 device (Clement Clarke, UK), which simulates the internal resistance of a MDI during inhalation. Participants were instructed to exhale fully to empty their lungs and then inhale as hard and fast as possible. Maximum peak PIF measurements were taken twice, being the highest value included in the analysis. PIF ≥ 30 L/min is considered the minimum requirement for the use of a DPI, and those who do not meet this requirement may need another type of inhaler [17].

The patient-reported level of knowledge regarding inhaler management was assessed by asking, without any guidance from the interviewer, whether their understanding was “good”, “fair”, or “poor”. The interview was conducted after the first week of hospitalization; if the patient was not present in the room after three attempts, they were considered absent.

To avoid altering routine clinical practice and to preserve the blinding of inhaled therapy assessments, the medical and nursing staff responsible for the patient’s hospitalization were not informed.

### 2.4. Data Analysis

Qualitative variables are presented as frequency distributions, while quantitative variables are summarized using the mean and standard deviation (SD). For quantitative variables with an asymmetric distribution, the median and interquartile range (IQR) are reported. Quantitative variables showing an asymmetric distribution are summarized with the median and interquartile range (IQR). For missing data, no imputation analysis was performed; possible confounding variables (such as age, gender, and duration of disease) will be adjusted in further analysis to ensure the robustness of the results. For the comparisons between the qualitative variables, the χ^2^ test or Fisher’s exact test was performed, as appropriate. For the comparisons of means between two independent groups, Student’s *t*-test were performed if the variables followed a normal distribution, and the non-parametric Mann–Whitney U test for asymmetric variables. The comparisons of means across more than two independent groups were performed using analysis of variance (ANOVA) or the non-parametric Kruskal–Wallis test for asymmetric variables.

A binary logistic regression model was used to explore the association between changing device at discharge and the other independent variables. Missing data were not imputed. A significant value of 5% was accepted for all tests. Data processing and analysis were performed using IBM SPSS Statistics v.26.

## 3. Results

### 3.1. Population Studied According to Inhaler Use History

#### 3.1.1. Patient Characteristic

A total of 499 and 474 patients with and without a previous history of inhaler use before hospitalization, respectively, were included in this study. Baseline patient characteristics are presented in Table 1.

The most frequent respiratory diseases in patients with an inhaled therapy prior to hospitalization were chronic obstructive pulmonary disease (COPD) in 53.7% and bronchial asthma in 17.8%. They also exhibited increased use of healthcare resources, as reflected by a higher proportion of patients with a history of at least one hospitalization in the previous year (77.4%) and those requiring two or more antibiotic cycles in the same period (46.5%). Some patients with respiratory comorbidities, such as COPD and asthma (6.8% and 3.4%, respectively), did not use at-home inhalers prior to hospitalization (Table 1).

#### 3.1.2. Inhaled Therapy Used and Reason

Among patients without prior inhaler use, the most common reasons for inhaler use during hospitalization were acute respiratory infections and heart failure (64.1% and 26.4% of patients, respectively). These patients were predominantly hospitalized in internal medicine and geriatrics services (58.9% and 33.8%, respectively) and exhibited higher in-hospital mortality compared to patients with a history of inhaler use at-home (20.5% vs. 3.6%). In contrast, 90-day post-discharge mortality was higher among patients with previous history of inhaler use and respiratory comorbidities (21.6% vs. 10.8%) (Table 1).

The most used inhaler devices during hospitalization, regardless of prior inhaler history, were MDIs with the valved holding chamber Aerochamber Plus^®^ (51.9% with previous inhaler use and 46.2% without previous inhaler use) and nebulizers (41.7% with previous inhaler use and 52.3% without previous inhaler use), with SABDs being the most frequently administered therapy (49.3% with previous inhaler use and 68.6% without previous inhaler use) (Table 1).

**Figure 2:**
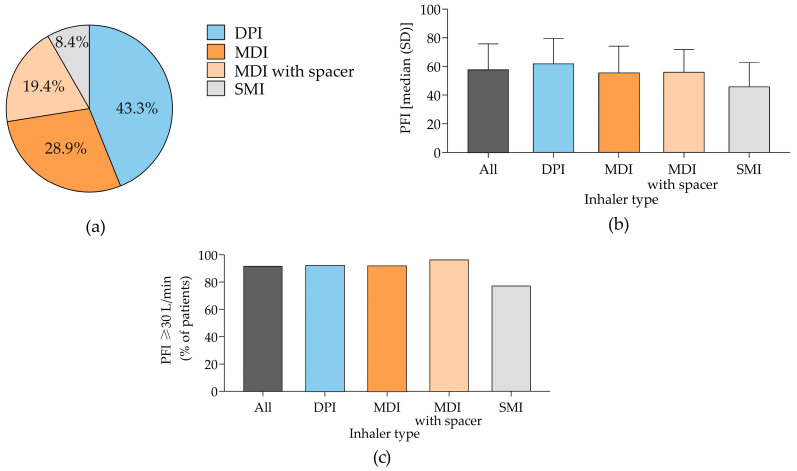
Characterization of patient population according to the type of inhalator used prior hospitalization. (**a**) Percentage of patients. (**b**) Median absolute maximum PIF values. (**c**) Percentage of patients with a maximum PIF ≥ 30 L/min. Panel (**a**) shows data from 499 patients, of whom 216, 144, 97, and 42 were using DPI, MDI, MDI with spacer, and SMI prior to hospitalization, respectively. Panel (**b**,**c**) shows data from 317 patients, of whom 144, 81, 70, and 22 were using DPI, MDI, MDI with spacer, and SMI prior to hospitalization, respectively. DPI, dry powdered inhaler; MDI, metered-dose inhaler; PIF, peak inspiratory flow; SMI, soft mist inhaler.

Among patients with a history of at-home inhaler use, less than half of the inhaled maintenance therapies in the community setting were dispensed as DPIs (43.3%) (Figure 2a). Approximately 70.1% of patients using MDI with a spacer were prescribed triple therapy [long-acting beta-agonist (LABA) + long-acting muscarinic antagonists (LAMA)+ inhaled corticosteroids (ICS)], with this treatment having the shortest duration of use (11 months) (Supplementary Table S1).

In our cohort, the overall median maximum PIF value was 57.7 L/min (Figure 2b), and most of patients (>90%) exhibited a maximum PIF ≥ 30 L/m, regardless of the type of inhaler used (Figure 2c).

### 3.2. Critical Inhaler Errors, Treatment Compliance, and Inhaler Handling-Related Knowledge in Patients with a Previous History of At-Home Inhaler Use

**Figure 3:**
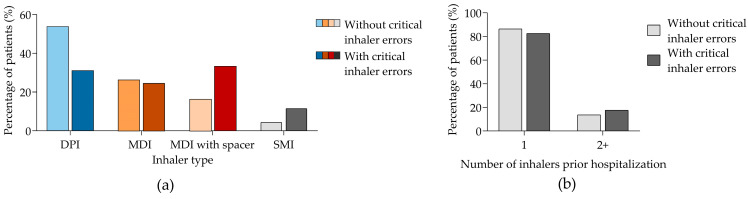
Percentage of patients according to performing inhaler critical errors or not. (**a**) By the type of inhaler used prior hospitalization; (**b**) by the number of inhalers used prior hospitalization. Panels (**a**,**b**) show data from 320 patients, of whom 206 and 114 were performing or not performing critical inhaler errors, respectively. DPI, dry powdered inhaler; MDI, metered-dose inhaler; SMI, soft mist inhaler.

The type of inhaler least associated with critical errors was the DPI (53.4%), whereas, conversely, the MDI with a spacer was associated with the highest rate of critical errors (33.3%). The highest technical error rate observed with MDI may be related to the relatively complex operation steps and the lack of patient guidance. More than half of patients using the MDI with spacer had critical errors (Figure 3a). Additionally, the number of devices used for inhaled therapy did not influence the occurrence of critical inhaler errors (86.4% without critical inhaler errors and 82.5% with critical inhaler errors for one inhaler; 13.6% and 17.5%, respectively, for two or more inhalers) (Figure 3b).

In our cohort, 65% of patients with critical inhaler errors had two or more critical errors. In the MDI with and without chamber and in the SMI devices, the most common critical inhaler errors detected were “not inhaling deeply and slowly” and “poor coordination between hand actuation and inhalation”. In addition, the activation of multiple doses in the chamber was a frequent error in more than 30% of patients with chamber errors. The most frequent DPI errors, observed in 48% and 30% of all patients with critical inhaler errors, were failure to hold breath for 5–10 s and failure to exhale before inhaling, respectively. This suggests that there is a need to strengthen patient education on inhaler techniques during hospitalization to improve treatment compliance and efficacy.

Nearly 70% of patients demonstrated poor or intermediate inhaler compliance, with the percentage of patients with poor compliance being higher among those using soft mist inhalers (SMIs; 53.3%) (Table 2). Independently of the type of inhaler used prior to hospitalization, most of the patients (95.4%) had unconscious noncompliance with the inhaler (Table 2).

Approximately one-third of patients with prior inhaler use had critical inhaler errors (35.6%). These patients had statistically significantly lower maximum PIF values (52.1 L/min in patients with critical inhaler errors vs. 60.8 L/min without critical inhaler errors; *p* > 0.001) and were more likely to exhibit poor inhaler compliance compared to those without critical errors (50.5% vs. 31.0%, respectively) (Table 3). Additionally, over 80% of patients without critical inhaler errors demonstrated good knowledge of inhaler handling, whereas 50.5% of patients with regular or poor knowledge committed critical inhaler errors (Table 3). Additional patient demographics and characteristics based on the presence or absence of critical inhaler errors are presented in Supplementary Table S2.

### 3.3. Inhaled Therapy Based on Maximum PIF Levels

In our cohort of patients with a history of at-home inhaler use, more than 90% demonstrated a maximum PIF ≥ 30 L/min (Table 4).

Older age and a higher burden of comorbidities were more frequent in patients with a maximum PIF < 30 L/min (Supplementary Table S3). However, despite these variables being more common in this population, no strong correlation was found between them (Supplementary Figure S1).

### 3.4. Patient Adherence to Inhaler Treatments

Among patients who had previously used at-home inhaled therapy, more than half showed regular or poor treatment adherence (69.9%), although 75.6% demonstrated good knowledge about inhaler handling. Interestingly, nearly 90% of patients with good treatment adherence used only one device (Table 5).

### 3.5. Change of Pre-Hospitalization Inhaled Therapy Device at Discharge

**Figure 4:**
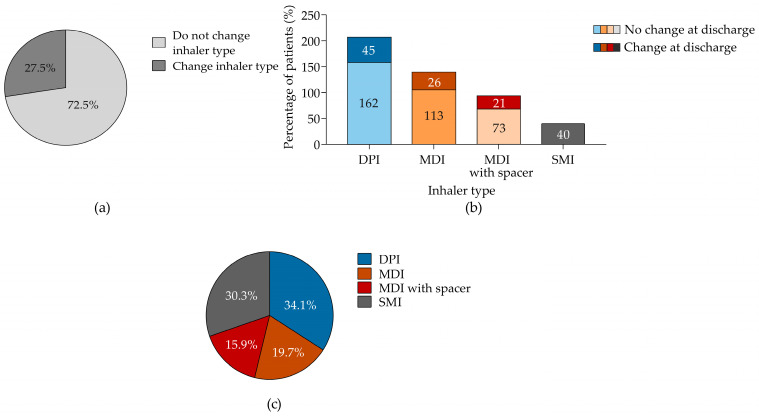
Patients who changed inhalers after hospitalization. (**a**) Percentage of patients who changed or did not change their inhaler. (**b**) Percentage of patients who changed or did not change according to the type of inhaler used prior to hospitalization. (**c**) Frequency of device changes at discharge according to the type of device used prior to hospitalization. Panel (**a**,**b**) shows data from 480 patients, of whom 132 and 348 changed or did not change inhaler type after hospitalization, respectively. DPI, dry powdered inhaler; MDI, metered-dose inhaler; SMI, soft mist inhaler.

Among inhaler users, only 27% of patients changed their inhaler type after hospitalization (Figure 4a). The highest percentage of inhaler changes was observed among DPI users (34.1%) (Figure 4c) and was associated with the type of therapy prescribed (LABA + ICS, LAMA + LABA + ICS) (Table 6). Our analysis did not identify any significant association between patient characteristics and the decision to change the inhaler type (Supplementary Table S4).

Overall, no significant differences were observed in inhaler changes when considering the frequency of critical inhaler errors, inhaler handling-related knowledge, or maximum PIF values (Table 7). More than half of patients (53.7%) who made critical inhaler errors while using an MDI with a spacer did not change their inhaler type at discharge (Table 7).

## 4. Discussion

By using real-world data from a clinical audit, this study provides novel insights about inhaled therapy in hospitalized patients, focusing on their at-home inhaled therapy before, during, and after hospital discharge Our analysis explored the clinical characteristics of patients, their knowledge of inhaler handling, critical inhaler errors, adherence rates, maximum PIF values, and changes in inhaler devices at discharge. Additionally, herein, we also explore the factors associated with inhaler changes at discharge.

In our cohort of patients, our main findings are that only less than a third of patients changed their prescribed inhaler device at hospital discharge, despite showing critical inhaler errors, reporting a poor level of handling-related knowledge about its use, and exhibiting low adherence to at-home inhaler treatment prior to hospitalization. This evidence highlights an important area for improvement in the use of inhalers among hospitalized patients with a high consumption of healthcare resources. Furthermore, our results point out the importance of evaluating patients’ inhaled therapies, assessing their individual characteristics to match the device to their needs, and providing patient education and training about proper inhaler use.

Respiratory diseases are a major health problem and a major contributor to pharmaceutical expenditure, with inhaler prescriptions ranking among the highest in total drug costs [18]. Despite the continuous innovations and the availability of numerous effective inhaled therapy options, inhaler misuse remains a significant challenge in managing chronic respiratory diseases, directly impacting disease outcomes [19,20]. Inhaled therapy is the cornerstone of respiratory disease management; however, the importance of selecting the most appropriate inhaler and the assessment of its correct use are still ignored or underestimated by some healthcare professionals [4,5]. The 2021 EPOCONSUL audit of pulmonology practice revealed that inhalation technique was evaluated in less than half of the audited visits in patients treated in the outpatient respiratory clinic [21]. Clinical practice guidelines underline the relevance of assessing inhalation technique during follow-up and management of inhaled therapy.

### 4.1. Inhaler Selection

Personalized inhaler selection and continuous monitoring by healthcare professionals are essential. PIF is the maximum flow rate achieved during an inspiratory maneuver, and its evaluation is an effective tool to help clinicians in selecting the most appropriate inhaler for each patient [17,22]. A maximum PIF of ≥30 L/min is considered to be sufficient for the effective use of most DPIs [17]. Thus, patients with a PIF ≥ 30 L/min are theoretically more likely to benefit from using a DPI, as this flow rate is sufficient for effective aerosolization and drug delivery to the lungs [17]. Studies have shown that the ability to generate a PIF of ≥30 L/min is independent of patient age or the severity of airway obstruction [17]. In the selection of the most appropriate inhaler, the determination of PIF values may be a valuable tool to improve treatment outcomes [23]. In our cohort, more than 90% of patients with a history of at-home inhaler use demonstrated a PIF ≥ 30 L/min, indicating they had adequate flow rates that may be considered adequate for optimal DPI performance. Although, to guarantee a correct drug administration in patients with respiratory diseases, it is crucial to consider the airflow resistance within different DPI devices and evaluate it for each patient, since the effects of airflow resistance differ between healthy subjects, asthmatics, and patients with COPD [24,25]. This airflow resistance variation between individuals highlights the importance of personalizing the choice of the DPI device based on an understanding of each device’s characteristics and evaluating the patient’s PIF in relation to the device [24,25]. However, in our population, half of patients used an MDI with or without a space despite being a device associated with higher rates of misuse and with a significant carbon footprint [10,20]. Also, in the hospital setting, regardless of the history of inhaler use, the MDI with spacer chamber was the most commonly used device. In clinical practice, there is an increasing need for more individualized inhaler selection to achieve optimal clinical outcomes. Routine PIF analysis can serve as a valuable and practical tool to guide this process, ensuring that the chosen inhaler is aligned with the patient’s needs and capabilities. Previous studies, such as that by Clark et al., have reported that 56.9% of patients hospitalized due to acute exacerbations of COPD showed low PIF values (<60 L/min) when measured with a medium–low resistance [26]. Also, a study conducted by Moon et al. found that 22% of COPD patients in consultation had devices inappropriate for their PIF measurements [27].

### 4.2. Critical Errors in Inhaled Therapies

In patients with chronic use of inhaled therapy, critical inhaler errors are directly associated with poor disease outcomes, including increased use of MDI rescue medications, reduced quality of life, higher rates of emergency department visits and hospitalizations, and greater dependence on oral steroids and antimicrobials [19,24,28,29,30]. In our cohort, we observed a high prevalence of critical inhaler errors, with one-third of patients demonstrating errors in inhaler handling. Our findings align with previous studies showing that inhalers are used incorrectly in 12–71% of cases [18]. In fact, more than half of patients using MDIs with chamber performed critical errors and 38% of patients using MDIs demonstrated poor inhaler technique compared to 23% of those using DPIs [12]. The most frequent error in DPI use was a failure to maintain apnea for 5–10 s. Interestingly, several studies have shown that errors in the steps of “inhale”, “hold your breath”, and “exhale calmly after inhalation” were significantly associated with poorer health status and a higher number of severe exacerbations [31]. However, very demanding specifications such as maintaining post-inhalation apnea for 5 s or 10 s, may be difficult targets in real life, although such critical errors have been correlated with higher COPD-related costs [32]. In this regard, these studies underline the importance of continuing education and regular, periodic review to improve the correct use of inhalers and achieve better clinical results [33]. DPIs, in comparison to MDI, are generally associated with fewer critical errors due to their breath-actuated mechanism, which eliminates the need to coordinate actuation with inhalation [10,23,24]. In the MDI with and without a chamber and in the SMI devices, the most common critical inhaler errors detected were “not inhaling deeply and slowly” and “poor coordination between hand actuation and inhalation”. In addition, the activation of multiple doses in the chamber was a frequent error in more than 30% of patients with chamber errors.

Moreover, patients using multiple types of inhalers are at a higher risk of critical errors. In contrast, the use of single and simple device inhalers, are often the best therapeutic option to minimize these errors and ensure the delivery of the drug dose during treatment [34]. Interestingly, in our cohort, nearly 90% of patients with good treatment adherence used only one device. A study also found that patients using multiple inhalers had a higher risk of exacerbations, as well as greater healthcare utilization and economic burden, compared to those using a single inhaler [35]. Thus, the use of multiple inhalers is associated with lower compliance, and patient-reported confusion regarding their inhaled medication has been identified as an independent predictor of non-adherence to treatment [36]. In our analysis, patients with critical inhaler errors were more likely to show poor inhaler compliance compared to those without critical errors (50.5% vs. 31%, respectively). Errors during this process can result in insufficient drug delivery to the lungs [8].

### 4.3. Compliance to Inhaled Treatment

Poor adherence and critical errors in inhaled therapies have previously been linked to an increased risk of rescue medication use and exacerbations, as well as higher mortality rates [30]. The proper use of an inhaler device requires careful preparation and handling prior to inhalation, along with an effective inhalation technique. Patient education and training are crucial to prevent critical errors in inhaler use, with a better knowledge of inhaler handling being associated with fewer errors. In our real-world cohort, in patients with a higher burden of healthcare utilization and higher post-discharge mortality, we observed that more than 50% of patients with regular or poor knowledge had critical inhaler errors. However, a good knowledge of inhaler handling does not necessarily predict good compliance, as some patients may continue to misuse their inhalers despite proper training [11]. In fact, while around 50% of patients in our study had good inhaler handling knowledge, more than 70% exhibited poor or intermediate compliance. The most common form of noncompliance was unconscious noncompliance, which aligns with previously reported evidence [37]. Despite a good understanding of inhaler techniques, many patients still exhibit suboptimal adherence to prescribed therapy [37]. Higher patient satisfaction with the inhaler, regardless of the medication received, correlates with better adherence [28,38,39]. Shared decision making during inhaler selection, in cooperation with patients, is the preferred approach to avoid critical inhaler errors, improve treatment compliance, and achieve optimal disease control [20].

In our cohort of patients, the most common method of inhaled drug administration during hospitalization was SABD via nebulization, either with oxygen or with a spacer. This aligns with previous studies that have evaluated inhaled therapies in the hospital setting to treat acute episodes, such as COPD exacerbations or respiratory infections [40]. This approach is driven by the need to use a device that facilitates drug delivery when the patient’s clinical condition is compromised. Additionally, the preferential use of nebulizers in hospitals compared to other devices may also be influenced by the availability of certain medications and inhaler models in the hospital pharmacy.

### 4.4. Clinical Inertia

Clinical inertia is defined as the failure to act when a patient does not achieve therapeutic objectives. These actions are not limited to initiating or intensifying therapy but also include evaluating potential aggravating factors, such as reviewing inhalation technique and assessing therapeutic adherence. When evaluating device changes at discharge in patients on inhaled therapy prior to hospitalization, we found that only 27% of patients changed their inhaler type after hospitalization. This is interesting because a considerable percentage of patients made critical inhaler errors with their inhalers and used an MDI with a chamber, despite having a maximum PIF that would allow them to transition to a more user-friendly DPI. These results are even more remarkable if we consider that this population consisted of hospitalized patients with a high level of intervention due to their history of prior hospitalizations and a high risk of readmission and mortality. The probability of hospital readmission after discharge is high [41]. In COPD, one in five patients were re-hospitalized within 30 days of discharge following an exacerbation admission [42]. It is estimated that approximately 50% of these readmissions could be avoided, as they often result from a fragmented healthcare system that provides inadequate discharge instructions, fails to educate patients about their therapy, and lacks effective communication with outpatient physicians responsible for follow-up care [43].

Our analysis did not identify any significant association between patient characteristics, critical inhaler errors, handling-related knowledge, or maximum PIF values and changes in inhaler type. The only factor associated with a change was the type of therapy prescribed, with the double (LABA + ICS) or triple therapy (LAMA + LABA + ICS) being associated with a higher likelihood of device change, suggesting that the reasons for switching inhaled devices are related to therapeutic adjustment strategies or changes in pharmacological treatment. One important aspect to consider when interpreting these results related to factors influencing device changes is that the assessment of compliance, technique, and PIF was conducted within the context of the study. The highest percentage of inhaler changes was observed among DPI users, although more than half of the patients who made critical inhaler errors while using an MDI with a spacer did not have their inhaler type changed at discharge. To avoid disrupting routine clinical practice and to maintain the blinding of inhaled therapy assessments, the medical and nursing staff responsible for the patient’s hospitalization were not informed

These findings suggest that clinicians possibly believe that the use of an inhalation chamber ensures a better drug delivery; although, our results show that these devices are often used with critical inhaler errors. Additionally, factors such as the patient’s PIF, inhaler technique (critical inhaler errors and adherence), and the patient’s level of knowledge about inhaler handling are frequently not considered in the decision to adjust treatment settings. This observation was highlighted by the results of the EPOCONSUL audit, which found that inhaled therapy is reviewed in less than half of follow-up visits [21].

These findings highlight the need for regular review of inhaled therapy during follow-up care, particularly after hospitalization. In-hospital training interventions (e.g., systematic assessment of inhaler technique and PIF, availability of tools as CHECK DIAL that facilitate device-patient adaptation) combined with selection of an appropriate inhaler and provision of therapy education, are identified as a valuable strategy to improve the impact of hospitalization on relevant outcomes like readmission and mortality [39]. Also, promoting nursing involvement in the assessment and education of inhaled therapy is a priority. Addressing inhalation technique errors and improving adherence requires a combination of continuous education, personalized device selection, and the integration of innovative technologies. The design of our study did not include follow-up of patients after discharge and only contemplate the collection of the pharmacotherapeutic patient history regarding the inhaled therapy prior hospitalization from the Single Prescription Module (MUP) program. However, we consider relevant to invest in future research to analyze whether the systematic characterization of the patient and the inhaler for adjusting inhaled therapy after hospitalization can lead to better outcomes in disease control and more efficient use of healthcare resources. Evidence supporting the development of specialized units and the role of nursing in reviewing inhaled therapy would be valuable in this regard.

### 4.5. Limitations of the Study

The main strength of this study lies in its provision of novel information. However, several considerations must be taken into account to accurately interpret our results. The primary limitation, common to any real-life study, is the presence of missing values (data not available), regardless of the inclusion methodology and regular monitoring of the database. In our study, a number of patients could not be assessed for the interview measures due to repeated absences. Another important consideration is that PIF measurement was performed in patients hospitalized during an unstable phase, which may have led to an underestimation of the PIF, as studies show that PIF can decrease during exacerbations. Additionally, this is a single-center study, and therefore, the results may not be representative of other populations. The present study did not have sufficient power to evaluate the influence of the different factors and their impact on the results, as it was a cross-sectional study. Despite these limitations, we believe the findings are consistent with those published on the use of inhaled therapy. Future clinical research should focus on evaluating the importance of ongoing education, personalized device selection using PIF measurement tools, and the training of healthcare professionals. Additionally, it should assess the prospective impact of guideline adherence on clinically relevant outcomes.

## 5. Conclusions

This study provides valuable information on inpatient inhaled therapy and changes in inhaler devices at discharge, exploring the associated determinants using real-world data. Therapeutic inertia is a key factor contributing to high hospital readmission rates and poor disease control following hospitalization. Our results showed the presence of therapeutic inertia, because despite the high rate of critical inhaler errors, only a small proportion of patients had their inhaler type changed after hospitalization. Our findings highlight the urgent need for personalized inhaler selection and routine monitoring to reduce misuse, improve adherence, and optimize disease management. Moreover, tackling this problem through a comprehensive regular assessment of inhalation technique upon discharge, promote simple, easy-to-use inhalers (such as DPIs) and provide regular education programs for patients may significantly improve clinical outcomes.

## Figures and Tables

**Table 1:** Summary of patient demographics and clinical characteristics according to the previous medical history of inhaled therapy.

	With Previous History of Inhaler Use Before Hospitalization	Without Previous History of Inhaler Use Before Hospitalization	*p*-Value
Patients included, n (%)	499 (51.3)	474 (48.7)	
Age, median (SD)	75.4 (12.4)	79.2 (12.7)	<0.001
Gender (men), n (%)	243 (59.6)	167 (49)	0.005
Current smoker, n (%)	54 (10.8)	25 (5.8)	0.381
Charlson index, median (SD)	3 (1–4)	2 (1–4)	0.002
Patients with Charlson index ≥ 2, n (%)	340 (67.9)	301 (63.5)	0.151
Respiratory comorbidities, n (%)			<0.001
Absence	66 (13.2)	361 (76.2)	
COPD	268 (53.7)	32 (6.8)	
Bronchiectasis	40 (8)	4 (0.8)	
Asthma	89 (17.8)	16 (3.4)	
Other	36 (7.2)	61 (12.9)	
Number of hospitalizations in previous year, median (IQR)	2 (1–3)	1 (0–2)	<0.001
Hospitalizations previous year ≥ 1, n (%)	388 (77.4)	271 (57.2)	<0.001
Antibiotic/corticosteroid courses in previous year, median (IQR)	1 (0–3)	0 (0–1)	<0.001
Number of courses ≥ 2, n (%)	232 (46.5)	103 (21.7)	<0.001
Cause for therapy during admission, n (%)			<0.001
COPD exacerbation	205 (40.9)	23 (4.9)	
Asthma exacerbation	17 (3.4)	5 (1.1)	
Bronchiectasis	10 (2)	0	
Respiratory infection	218 (43.5)	304 (64.1)	
Cardiac insufficiency	43 (8.6)	125 (26.4)	
Inpatient service, n (%)			<0.001
Internal medicine	260 (52.1)	279 (58.9)	
Pulmonology	125 (25)	35 (7.4)	
Geriatrics	114 (22.8)	160 (33.8)	
Inhaled therapy during hospitalization, n (%)			0.434
SABD	246 (49.3)	325 (68.6)	
ICS + SABD	96 (19.2)	67 (14.1)	
LAMA	30 (6)	14 (3)	
LABA + LAMA	17 (3.4)	5 (1.1)	
LABA + ICS	35 (7)	27 (5.7)	
LABA + LAMA + ICS (single inhaler)	66 (13.2)	27 (5.7)	
LABA + LAMA + ICS (multiple inhalers)	9 (3.4)	9 (1.9)	
Inhaler devices during hospitalization, n (%)			0.212
MDI (with spacer)	260 (51.9)	219 (46.2)	
Nebulizer	209 (41.7)	248 (52.3)	
MDI	2 (0.4)	1 (0.2)	
DPI	14 (2.8)	1 (0.2)	
SMI	13 (2.6)	5 (1.0)	
Mortality, n (%)			<0.001
In-hospital	18 (3.6)	97 (20.5)	
90 days	108 (21.6)	51 (10.8)	

%, percentage; COPD, chronic obstructive pulmonary disease; CS, corticosteroids; DPI, dry powdered inhaler; ICS, inhaled corticosteroids; IQR, interquartile range; LABA, long-acting beta-agonist; LAMA, long-acting muscarinic antagonists; MDI, metered-dose inhaler; SABD, short-acting bronchodilators; SD, standard deviation; SMI, soft mist inhaler.

**Table 2:** Adherence to inhaled therapy prior hospitalization.

	All Patients	Patients Using DPI	Patients Using MDI	Patients Using MDI with Spacer	Patients Using SMI
Patients included, n (%)	279 (100)	135 (48.4)	68 (24.4)	61 (21.9)	15 (5.3)
Inhaled therapy compliance, n (%)					
Poor	106 (38)	51 (37.8)	24 (35.3)	23 (37.7)	8 (53.3)
Intermediate	89 (31.9)	47 (34.8)	23 (33.8)	16 (26.2)	3 (20)
Good	84 (30.1)	37 (27.4)	21 (30.9)	22 (36.1)	4 (26.7)
Type of inhaler noncompliance, n (%)					
Erratic	165 (59.1)	76 (56.3)	43 (63.2)	35 (57.4)	11 (73.3)
Deliberate	35 (12.5)	9 (6.6)	7 (10.3)	13 (21.3)	6 (40)
Unconscious	266 (95.4)	126 (93.3)	66 (97.0)	60 (98.3)	14 (93.3)

%, percentage; DPI, dry powdered inhaler; MDI, metered-dose inhaler; SMI, soft mist inhaler.

**Table 3:** Patient compliance and knowledge regarding inhaler handling technique in patients with/without critical errors.

	Without Critical Inhaler Errors	With Critical Inhaler Errors	*p*-Value
PIF, median (SD)	60.8 (17.3)	52.1 (17.8)	<0.001
Inhaler compliance, n/N (%)			0.007
Poor	57/184 (31)	46/91 (50.5)	
Intermediate	66/184 (35.9)	22/91 (24.2)	
Good	61/184 (33.2)	23/91 (25.3)	
Inhaler handling-related knowledge, n/N (%)			<0.001
Good	172/195 (88.2)	49/99 (49.5)	
Regular or poor	23/195 (11.8)	50/99 (50.5)	

n values represent the number of patients that meet a specific criteria and N the total number of patients considered in the analysis. %, percentage; PIF, peak inspiratory flow.

**Table 4:** Inhaled therapy prior hospitalization in patients with a maximum PIF < 30 L/min or ≥30 L/min.

	PIF ≥ 30 L/min	PIF < 30 L/min
**Inhaler device, n/N (%)**		
DPI	133/294 (45.2)	11/27 (40.7)
MDI	74/294 (25.2)	7/27 (25.9)
MDI with spacer	67/294 (22.8)	3/27 (11.1)
SMI	17/294 (6.8) 5.8	5/27 (18.5)
Unknown	3/294 (1.0)	1/27 (3.7)
Number of inhalers, n/N (%)		
1	252/294 (85.7)	22/27 (81.5)
2	42/294 (14.3)	5/27 (18.5)
Treatment period, median (IQR)	15 (7–40)	25 (7–63)
Patients with critical inhaler errors, n/N (%)	96/294 (32.6)	18/27 (66.7)
Type of inhaler compliance, n/N (%)		
Poor	98/253 (38.7)	8/24 (33.3)
Intermediate	84/253 (33.2)	5/24 (20.8)
Good	71/253 (28.1)	11/24 (45.8)
Inhaler handling-related knowledge, n/N (%)		
Good	212/272 (77.9)	9/24 (37.5)
Regular or poor	60/272 (22.1)	15/24 (62.5)

n values represent the number of patients that meet a specific criteria and N the total number of patients considered in the analysis. %, percentage; DPI, dry powdered inhaler; MDI, metered-dose inhaler; PIF, peak inspiratory flow; SMI, soft mist inhaler.

**Table 5:** Factors related to patients’ adherence to prescribed inhaler treatments.

	Good Inhaler Treatment Adherence	Regular/Poor Inhaler Treatment Adherence	*p*-Value
Inhaler device, n/N (%)			0.512
DPI	37/84 (44)	98/195 (50.3)	
MDI	21/84 (25)	47/195 (24.1)	
MDI with spacer	22/84 (26.2)	39/195 (20)	
SMI	4/84 (4.8)	11/195 (5.6)	
Number of inhalers, n/N (%)			0.052
1	74/84 (88.1)	166/195 (8.1)	
2	10/84 (11.9)	29/195 (14.9)	
Patients with maximum PIF ≥ 30 L/min, n/N (%)	70/84 (86.4)	180 (93.8)	0.868
Patients with critical inhaler errors, n/N (%)	23/84 (27.4)	68/195 (34.9)	0.182
Inhaler handling-related knowledge, n/N (%)			0.099
Good	71/84 (84.5)	146/193 (75.6)	
Regular or poor	13/84 (15.5)	47/193 (24.4)	

n values represent the number of patients that meet a specific criteria and N the total number of patients considered in the analysis. %, percentage; DPI, dry powdered inhaler; MDI, metered-dose inhaler; PIF, peak inspiratory flow; SMI, soft mist inhaler.

**Table 6:** Factors associated with not changing inhaler type after hospitalization.

	OR	95% CI	*p*-Value
LAMA (ref)	1	-	-
LAMA + LABA	0.876	0.422–1.817	0.722
LABA + ICS	0.391	0.182–0.838	0.016
LABA + LAMA + ICS	0.369	0.178–0.764	0.007
1 single inhaler	1.817	0.549–6.011	0.328

CI, confidence interval; OR, odds ratio; Ref, reference.

**Table 7:** Patients with critical inhaler errors. PIF values and inhaler handling-related knowledge according to changing or not inhaler type after hospitalization.

	Do Not Change Inhaler Type	Change Inhaler Type		
	**DPI Prior Hospitalization**	**DPI Prior Hospitalization**		
		**Change to MDI**	**Change to MDI with Spacer**	**Change to SMI**
Patients included, n/N	162/348 (46.6)	35/45 (77.8)	10/45 (22.2)	0/45 (0)
Patients with critical inhaler errors, n/N (%)	28/110 (25.5)	6/35 (22.2)	1/10 (14.3)	-
Patients with maximum PIF < 30 L/min, n/N (%)	10/160 (6.2)	1/35 (3.7)	0/10	-
Regular or poor inhaler handling-related knowledge, n (%)	16/106 (15.1)	4/35 (13.8)	0/10	-
	**MDI prior hospitalization**	**MDI prior hospitalization**		
		**Change to DPI**	**Change to MDI with spacer**	**Change to SMI**
Patients included, n/N	113/348 (32.5)	14/26 (53.9)	11/26 (42.3)	1/26 (3.8)
Patients with critical inhaler errors, n/N (%)	22/64 (34.4)	0 (0)	4/11 (50)	0
Patients with PIF <30 L/min, n/N (%)	5/63 (7.9)	0 (0)	1/11 (12.5)	0
Regular or poor inhaler handling-related knowledge, n (%)	21/61 (34.4)	1/14 (16.7)	4/11 (50)	1 (100)
	**MDI with spacer prior hospitalization**	**MDI with spacer prior hospitalization**		
		**Change to DPI**	**Change to MDI**	**Change to SMI**
Patients included, n/N	73/348 (21.0)	8/21 (38.1)	13/21 (61.9)	0/21
Patients with critical inhaler errors, n/N (%)	29/54 (53.7)	2/7 (28.6)	6/9 (66.7)	-
Patients with PIF <30 L/min, n/N (%)	3/54 (5.6)	0 (0)	0 (0)	-
Regular or poor inhaler handling-related knowledge, n (%)	17/49 (34.7)	1/6 (16.7)	2/6 (33.3)	-
	**SMI prior hospitalization**	**SMI prior hospitalization**		
		**Change to DPI**	**Change to MDI**	**Change to MDI with spacer**
Patients included, n/N	**-**	13/40 (32.5)	6/40 (15.0)	21/40 (52.5)
Patients with critical inhaler errors, n/N (%)	-	4/8 (50)	1/3 (33.3)	8/11 (72.7)
Patients with PIF < 30 L/min, n/N (%)	-	1/8 (12.5)	1/3 (33.3)	3/11 (27.3)
Regular or poor inhaler handling-related knowledge, n (%)	-	3/8 (37.5)	0 (0)	4/7 (57.1)

n values represent the number of patients that meet a specific criteria and N the total number of patients considered in the analysis. %, percentage; DPI, dry powdered inhaler; MDI, metered-dose inhaler; PIF, peak inspiratory flow; SMI, soft mist inhaler.

## Data Availability

The original contributions presented in the study are included in the article; further inquiries can be directed to the corresponding authors. The data presented in this study are available on request from the corresponding author.

## Supplementary Materials

The following supporting information can be downloaded at: https://www.mdpi.com/article/10.3390/jcm14041217/s1, Table S1. Patient demographics and clinical characteristics according to the type of inhaler used prior hospitalization; Table S2. Patient demographics and clinical characteristics according to performing inhaler critical errors or not; Table S3. Patient demographics and clinical characteristics according to PIF levels at hospitalization; Table S4. Patient demographics and clinical characteristics according to changing or not changing inhaler type after hospitalization; Figure S1. Correlation of age (a) and Charlson index (b) with PIF in patients with prior inhalers before hospitalization.
