# Supplementary material for: Insights from Real-World Evidence on the Use of Inhalers in Clinical Practice
Source: J Clin Med. 2025 Feb 12;14(4):1217. doi: 10.3390/jcm14041217 (PMC11856687; doi:10.3390/jcm14041217)
Supplement: Supplementary file 1 [file jcm-14-01217-s001.zip › jcm-3399641-supplementary.pdf]

Table S1. Patient demographics and clinical characteristics according to the type of inhaler used prior hospitalization

	All patients	Patients using DPI	Patients using MDI	Patients using MDI with spacer	Patients using Respimat
**Patients included, n (%)**	499 (100)	216 (43.3)	144 (28.9)	97 (19.4)	42 (8.4)
**Age, median (SD)**	75.4 (12.4)	75.1 (12.7)	75.5 (12.1)	76.8 (11.8)	74.7(14.0)
**Gender (men), n (%)**	243 (59.6)	106 (60.9)	63 (55.3)	52 (62.7)	22 (59.5)
**Respiratory comorbidities, n (%)**					
Absence	66 (13.2)	32 (14.8)	18 (12.5)	8 (8.2)	8 (19)
Asthma	89 (17.8)	38 (17.6)	31 (21.5)	15 (15.5)	5 (11.9)
COPD	268 (53.7)	110 (50.9)	73 (50.7)	67 (69.1)	18 (42.9)
Other	76 (15.2)	36 (16.7)	22 (15.2)	7 (7.2)	11 (26.2)
**Hospitalizations previous year ≥1, n (%)**	386 (77.4)	159 (73.6)	115 (79.9)	82 (84.5)	30 (71.4)
**Inhaled therapy, n (%)**					
LAMA/LABA	51 (10.3)	28 (13)	5 (3.5)	3 (3.1)	15 (35.7)
LABA+LAMA	101 (20.3)	64 (29.8)	19 (13.3)	0	18 (42.9)
LABA+ICS	118 (23.7)	57 (26.5)	40 (28)	21 (21.6)	0
LABA+LAMA+ICS (single inhaler)	160 (32.2)	45 (20.9)	47 (32.9)	68 (70.1)	0
LABA+LAMA+ICS (multiple inhalers)	67 (13.5)	21 (9.8)	32 (22.4)	5 (5.2)	9 (21.4)
**Treatment period before hospitalization, median (IQR)**	17 (7 – 45)	26 (10-63.5)	18.5 (6.25-36)	11 (6-16.5)	15.5 (10-36)
**Trainer, n (%)**					
None	52 (17.2)	13 (9.1)	21 (26.3)	12 (19)	6 (37.5)
Nurse assistant (PC)	8 (2.6)	1 (0.7)	5 (6.3)	1 (1.6)	1 (6.3)
Nurse assistant (hospital)	46 (15.2)	17 (11.9)	15 (18.8)	11 (17.5)	3 (18.8)
Doctor (PC)	30 (9.9)	18 (12.6)	6 (7.5)	6 (9.5)	0
Doctor (specialist)	100 (33.1)	44 (30.8)	24 (30)	28 (44.4)	4 (25)
Pharmacy	66 (21.9)	50 (35)	9 (11.3)	5 (7.9)	2 (12.5)

%, percentage; COPD, chronic obstructive pulmonary disease; DPI, dry powdered inhaler; IQR, interquartile range; LABA, long-acting beta-agonist; LAMA, long-acting muscarinic antagonists; PC, primary care; SD, standard deviation.

Table S2. Patient demographics and clinical characteristics according to performing inhaler critical errors or not

	Without critical inhaler errors	With critical inhaler errors	p-value
**Patients included, n (%)**	206 (64.4)	114 (35.6)	
**Age, median (SD)**	75.2 (11.7)	74.9 (14.1)	0.898
**Gender (men), n (%)**	108 (63.2)	50 (48.5)	0.018
**Current smoker, n (%)**	15(7.3)	11 (9.6)	0.393
**Charlson , median (SD)**	3 (1-4)	2 (1-4)	0.116
**Patients with Charlson index ≥2, n (%)**	142 (68.9)	69 (60.5)	0.129
**Respiratory comorbidities, n (%)**			0.129
Absence	15 (7.3)	10 (8.8)	
Asthma	36 (17.5)	23 (20.2)	
COPD	122 (59.2)	62 (54.4)	
Other	33 (11.2)	19 (16.7)	
**Number of hospitalizations in previous year, median (IQR)**	2 (1-3)	2 (1-3)	0.525
**Hospitalizations previous year ≥1, n (%)**	165 (80.1)	87 (76.3)	0.428
**Responsible service, n (%)**			0.589
Pulmonology	50 (24.3)	29 (25.4)	
Internal medicine	104 (50.5)	62 (54.4)	
Geriatrics	52 (25.2)	23 (20.2)	
**Treatment period before hospitalization, median (IQR)**	18 (7.7 – 45.2)	14.5 (6.7 -38.5)	0.324
**Trainer (service), n (%)**			-
None	21 (10.7)	31 (31.3)	
PC	24 (13)	18 (21.7)	
Specialized	113 (71.5)	50 (60.2)	
Pharmacy	48 (25.9)	15 (18.1)	

%, percentage; COPD, chronic obstructive pulmonary disease; IQR, interquartile range; PC, primary care; SD, standard deviation.

Table S3. Patient demographics and clinical characteristics according to maximum PIF levels at hospitalization

	PIF <30 L/min	PIF ≥30 L/min
**Patients included, n (%)**	294 (91.6)	27 (8.4)
**Maximum PIF, median (SD)**	60.6 (15.6)	24.7 (7.6)
**Age, median (SD)**	74.29 (12.56)	84.26 (8.89)
**Gender (men), n %**	147 (50)	12 (44.4)
**Current smoker, n %**	26 (8.8)	0
**Charlson index, median (SD)**	2 (1-4)	3 (1-5)
**Patients with Charlson index ≥2, n %**	194 (66)	19 (70.4)
**Respiratory comorbidities, n %**		
Absence	21 (7.1)	3 (11.1)
Asthma	53 (18)	5 (18.5)
COPD	172 (58.5)	12 (44.4)
Other	48 (16.4)	7 (25.9)
**Number of hospitalizations in previous year, median (IQR)**	2 (1-3)	1 (0-3)
**Hospitalizations previous year ≥1, n (%)**	234 (79.6)	19 (70.4)
**Moderate exacerbations in previous year, median (IQR)**	1.5 (0-3)	1 (0-2)
**Antibiotic/corticosteroid courses in previous year ≥2, n (%)**	147 (50)	10 (37)
**Responsible service, n (%)**		
Pulmonology	78 (26.5)	1 (3.7)
Internal medicine	151 (51.4)	15 (55.6)
Geriatrics	65 (22.1)	11 (40.7)
**Cause for therapy during admission, n (%)**		
COPD	131 (44.6)	8 (29.6)
Asthma	11 (3.7)	2 (7.4)
Respiratory infection	127 (43.2)	14 (51.9)
Cardiac insufficiency	25 (8.5)	3 (11.1)
**At-home inhaled therapy, n (%)**		
LAMA/LABA	25 (8.5)	4 (14.8)
LABA+LAMA	60 (20.4)	3 (11.1)
LABA+ICS	73 (24.8)	9 (33.3)
LABA+LAMA+ICS (single inhaler)	100 (34.0)	6 (22.2)
LABA+LAMA+ICS (multiple inhalers)	36 (12.2)	5 (18.5)

%, percentage; COPD, chronic obstructive pulmonary disease; ICS, inhaled corticosteroids; IQR, interquartile range; LABA, long-acting beta-agonist; LAMA, long-acting muscarinic antagonists; SD, standard deviation.

Table S4. Patient demographics and clinical characteristics according to changing or not inhaler type after hospitalization

	Change inhaler type	Do not change inhaler type	p-value
**Patients included, n (%)**	348 (72.5)	132 (27.5)	
**Age, median (SD)**	76.1 (11.9)	74.4 (13.9)	0.196
**Gender (men), n (%)**	173 (60.5)	61 (58.7)	0.743
**Current smoker, n (%)**	36 (10.3)	16 (12.1)	0.479
**Charlson, median (SD)**	3 (1-4)	3 (1-4)	0.586
**Patients with Charlson index ≥2, n (%)**	238 (68.4)	85 (64.4)	0.405
**Respiratory comorbidities, n (%)**			0.321
Absence	34 (9.8)	17 (12.9)	
Asthma	66 (19)	22 (16.7)	
COPD	199 (57.2)	66 (50)	
Other	49 (14.1)	27 (20.4)	
**Cause for therapy during admission, n (%)**			0.565
COPD exacerbation	155 (44.5)	48 (36.4)	
Asthma exacerbation	12 (3.4)	4 (3)	
Respiratory infection	156 (44.8)	68 (51.5)	
Cardiac insufficiency	25 (7.2)	12 (9.1)	
**Responsible service, n (%)**			0.248
Pulmonology	81 (23.3)	40 (30.3)	
Internal medicine	188 (54)	62 (47)	
Geriatrics	79 (22.7)	30 (22.7)	
**Inhaled therapy prior hospitalization, n (%)**			<0.001
LAMA/LABA	28 (8.1)	17 (12.9)	
LABA+LAMA	65 (18.8)	35 (26.5)	
LABA + CSI	91 (26.3)	22 (16.7)	
LABA+LAMA+ICS (single inhaler)	126 (36.4)	29 (22)	
LABA+LAMA+ICS (multiple-inhalers)	36 (10.4)	29 (22)	
**Number of inhalers prior hospitalization, n (%)**			0.001
1	306 (87.9)	100 (75.8)	
2	42 (12.1)	32 (24.2)	
**Patients with critical inhaler errors, n (%)**	79 (34.6)	32 (36.8)	0.723
**Type of inhaler compliance, n (%)**			0.591
Poor	79 (38.7)	26 (37.1)	
Intermediate	63 (30.9)	26 (37.1)	
Good	62 (30.4)	18 (25.7)	
**Maximum PIF, median (SD)**	58.82 (18.22)	55.22 (16.55)	0.097
**Patients with maximum PIF ≤30 L/min, (%)**	18 (8)	7 (8.1)	
**Inhaler handling-related knowledge, n/N**			0.956
**(%)**	162/216 (75)	59/79 (74.7)	
Good	54/216 (25)	20/79 (25.3)	
Regular or poor			

n values represent the number of patients that meet a specific criteria and N the total number of patients considered in the analysis.%, percentage; COPD, chronic obstructive pulmonary disease; ICS, inhaled corticosteroids; LABA, long-acting beta-agonist; LAMA, long-acting muscarinic antagonists; SD, standard deviation; PIF, peak inspiratory flow.

Figure S1. Correlation between age (a) and Charlson index (b) with maximum PIF in patients with prior inhaler before hospitalization. PIF, peak inspiratory flow.

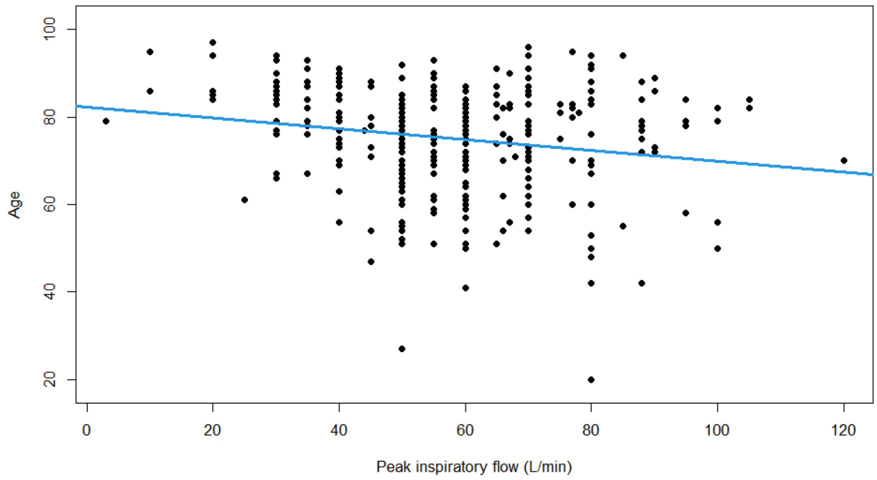

(a)

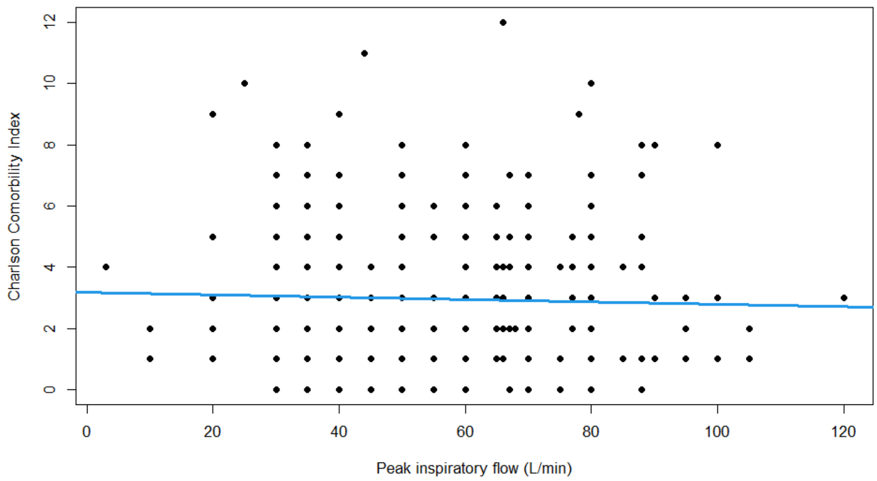

(b)
